# Investigation of the improvement effect of Dajianzhong decoction on rats with post-operative ileus based on serum metabolomics and spectrum-effect relationship

**DOI:** 10.3389/fphar.2026.1793033

**Published:** 2026-04-14

**Authors:** Zhufeng Cong, Tianren Xu, Xuan Kang, Zheng Zhong, Honglei Zhou, Yanan Li

**Affiliations:** 1 College of Pharmacy, Shandong University of Traditional Chinese Medicine, Jinan, China; 2 Shandong First Medical University Affiliated Tumor Hospital, Jinan, China; 3 Institute of Pharmacy, Shandong University of Traditional Chinese Medicine, Jinan, China; 4 Shandong Key Laboratory of Digital Traditional Chinese Medicine, Shandong University of Traditional Chinese Medicine, Jinan, China

**Keywords:** 5-HT modelation, Dajianzhong decoction, posroperative ileus, spectrum-effect relationship, untargeted metabolism

## Abstract

**Introduction:**

Postoperative ileus (POI) is a common complication following abdominal surgery, with limited preventive and therapeutic strategies. Dajianzhong Decoction (DJZD), a classic formula in traditional Chinese medicine, has demonstrated clinical potential in alleviating POI, yet its underlying composition and molecular mechanisms remain to be systematically elucidated.

**Methods:**

HPLC fingerprinting was performed on 30 batches of DJZD, and spectrum-effect correlation analysis was conducted by correlating fingerprint data with 5-HT secretion levels in RIN-14B cells. A rat POI model was established to compare the effects of preoperative preventive and postoperative therapeutic administration of DJZD on gastrointestinal motility. Serum untargeted metabolomics was employed to identify differentially expressed metabolites and analyze relevant metabolic pathways.

**Results:**

Eleven key metabolite from DJZD were screened and confirmed, namely, ginsenoside Rg_1_, ginsenoside Re, γ-Sanshool, 10-gingerol, ginsenoside Rb1, ginsenoside Rc, ginsenoside Rb3, ginsenoside Rb2, ginsenoside Rd, hydroxy-α-sanshool and hydroxy-β-sanshool. Among these conmponents, ginsenoside Rc and ginsenoside Rd were identified as the primary metabolites associated with DJZD’s regulation of 5-HT secretion. Both preoperative and postoperative administration of DJZD significantly improved gastrointestinal motility in POI rats, with preoperative intervention showing more pronounced effects. Metabolomic analysis revealed that DJZD modulated several key pathways, including tryptophan metabolism, steroid hormone biosynthesis, and glycerophospholipid metabolism.

**Conclusion:**

DJZD alleviates POI through multi-component, multi-pathway mechanisms involving the modulation of intestinal 5-HT signaling and systemic metabolic reprogramming. This study provides a pharmacological basis for the clinical use of DJZD in POI and exemplifies the holistic regulatory characteristics of traditional Chinese medicine.

## 1 Introduction

Postoperative ileus (POI) is a common complication after abdominal surgery, defined as non-mechanical gastrointestinal dysfunction (Kronberg et al., 2011). Its reported incidence following colorectal surgery ranges from 3% to 32% (Gero et al., 2017). Clinical features include abdominal distension, tenderness, and absent bowel sounds, with severe cases potentially leading to life-threatening outcomes (Zhong et al., 2023). POI prolongs hospitalization and heightens care demands in gastrointestinal surgical patients, while also raising the risk of other postoperative complications such as infection and thrombosis. Without timely prevention and intervention, POI may result in serious morbidity or even mortality. Consequently, effective prevention and management of POI are crucial to shorten hospital stays, alleviate patient discomfort, and improve postoperative quality of life, representing an urgent clinical priority.

Dajianzhong Decoction (DJZD) is a traditional formula for warming the middle burner, tonifying deficiency, descending adverse qi, and alleviating pain. It is composed of four botanical drugs: *Zanthoxylum bungeanum* Maxim. (HuaJiao), *Zingiber officinale* Rosc. (GanJiang), *Panax ginseng* C.A. Mey. (Renshen), and maltose syrup (Mao, 2004; He et al., 2025). Clinically, DJZD is widely applied in treating gastrointestinal disorders, including postoperative abdominal conditions. It has demonstrated safety and efficacy in early-stage simple intestinal obstruction without significant adverse reactions (Cheng, 2004). In Japan, it is a first-line agent for postoperative ileus, reducing the need for surgical adhesiolysis, and is also used for gastric ulcers, intestinal adhesions, biliary colic, and pancreatitis (Li, 1998). Clinical studies report its utility in functional constipation and abdominal distension (Li, 2009); combined with herbal enemas and acupoint patches, it significantly improves abdominal distension in patients with cold-deficiency intestinal obstruction (Fang and Xu, 2022). A UK study found that DJZD reduces blood ammonia levels and alleviates abdominal distension and diarrhea after liver resection (Yi, 2007). Furthermore, DJZD combined with conventional therapy improves outcomes in gastric ulcers attributed to spleen-stomach yang deficiency (Ren, 2018).

The efficacy of Kampo Daikenchuto (DKT) in treating POI is well-established. Research indicates that Dajianzhong Decoction significantly prevents intestinal obstruction in patients undergoing laparoscopic surgery for colorectal cancer, with its intervention shortens the duration of illness for patients (Kunitomi et al., 2023). Intestinal motility is closely linked to serotonin (5-HT) metabolism. Dysregulation of 5-HT synthesis, release, or signaling can disrupt motility and contribute to conditions like irritable bowel syndrome and POI (Eguchi et al., 2021). Given that impaired intestinal motility is central to POI and is modulated by mucosal 5-HT, investigating DJZD’s effects on intestinal motility and 5-HT expression may elucidate its therapeutic mechanism.

Enterochromaffin cells (ECs) are key intestinal epithelial chemosensors (Furness et al., 2013) that form synaptic connections with neurons (Yang et al., 2018; Dockray, 2013). They regulate nutrient absorption (Oguri et al., 2021; Yang et al., 2016), intestinal immunity (Linan-Rico et al., 2016), and are implicated in disorders such as irritable bowel syndrome (Mawe and Hoffman, 2013). ECs produce about 90% of the body’s 5-HT, which modulates gastrointestinal motility, immune responses, and visceral sensitivity (Xu et al., 2021).

Due to the difficulty of culturing primary ECs, the rat pancreatic endocrine cell line RIN-14B is often used as a model. RIN-14B cells secrete 5-HT (Reigstad et al., 2015), express EC markers such as chromogranin A and VMAT1 (Lyu et al., 2022), and have been employed in studies on colorectal cancer and postoperative ileus (Tsuchiya et al., 2016). Thus, they provide a valid *in vitro* model for EC physiology and pharmacology.

In this study, RIN-14B cells were used as an EC model. The fingerprint profiles of 30 batches of DJZD were correlated with their effects on 5-HT secretion in RIN-14B cells via spectrum-effect analysis, aiming to identify active metabolites responsible for its efficacy. Subsequently, serum untargeted metabolomics was employed to identify differential metabolites between preemptive intervention, postoperative treatment, and POI model rats, revealing relevant metabolic pathways to clarify the mechanism of DJZD in treating postoperative ileus.

## 2 Materials and methods

### 2.1 Materials and reagents

Analytical research (AR)-grade ethanol, ammonia solution, n-butanol, etc., were obtained from Tianjin Damao Chemical Reagent Co., Ltd. (Tianjin, China). Chromatography-grade methanol and acetonitrile were purchased from Merck KGaA (Darmstadt, Germany). Chromatography-grade phosphoric acid, glacial acetic acid, formic acid, etc., were purchased from ThermoFisher Scientific Inc. (United States). Wahaha purified water was obtained from Hangzhou Wahaha Group Co., Ltd. (China, Zhejiang). AL-204 0.0001  g balance and AB135-S 0.0001  g balance purchased from Mettler Toledo Instruments Co., Ltd. (Shanghai, China); KQ-500E ultrasonic extractor purchased from Kunshan Ultrasonic Instrument Co., Ltd. (Jiangsu, China); LC 2030A high-performance liquid chromatograph purchased from Shimadzu Corporation (Sichuan, China). The PK-S24 electric heating constant-temperature water bath was purchased from Shanghai Jinghong Laboratory Equipment Co., Ltd. (China, Shanghai). The MZ 2C NT vacuum pump was purchased from vacuubrand (China, Shanghai). RE 5298A rotary evaporator purchased from Shanghai Yarong Biochemical Instrument Factory (China, Shanghai), RIN-14B cells (Shanghai Yaji Biotechnology Co., Ltd.), Rat 5-Hydroxytryptamine ELISA Detection Kit (Enzyme Immunoassay Biotechnology Co., Ltd.), CCK8 Kit (Shanghai Biyuntian). Fluorescence microscope (Olympus, Japan).

The reference standards used are as follows: Ginsenoside Rb_1_ (110704–201726, 91.1%), Ginsenoside Re (110754–201626, 97.4%), Ginsenoside Rg_1_ (110703–202034, 94.0%), 6-Gingerol (111833–202007, 99.3%) purchased from China National Institute for Food and Drug Control (Beijing, China); Hydroxy-α-piparitan (83883–10–7, 98%), Hydroxy-β-piparitan (97465–69–5, 98%), Ginsenoside Rb_2_ (11021–13–9, 98%), Ginsenoside Rb_3_ (64806–26–8, 97%), Ginsenoside Rc (11021–14–10, 98%), Ginsenoside Rd (52705–93–8, 98%), Ginsenoside Rf (52286–58–5, 97%) were supplied by Shanghai Yuanye Biotechnology Co., Ltd. (Shanghai, China). ZBM, ZOR, GRR, and syrup were supplied by Shanghai Pharmaceutical Group Qingdao Guofeng Pharmaceutical Co., Ltd. and authenticated by Shandong pharmaceutical expert Song Xigui (Jinan Institute for Drug Control). Thirty batches of DJZD samples (designated DJZD1–DJZD30) were randomly formulated from three Chinese herbal medicines. Source and batch information for the herbal materials are listed in Table 1.

**TABLE 1:** Origin of 30 Batches of DJZD botanical drugs.

Baches	GRR	GOR	ZBM
DJZD-1	R-1	G-2	H-1
DJZD-2	R-1	G-2	H-2
DJZD-3	R-1	G-2	H-3
DJZD-4	R-2	G-2	H-4
DJZD-5	R-2	G-2	H-5
DJZD-6	R-2	G-2	H-6
DJZD-7	R-3	G-2	H-7
DJZD-8	R-3	G-1	H-8
DJZD-9	R-3	G-1	H-9
DJZD-10	R-4	G-1	H-10
DJZD-11	R-4	G-1	H-1
DJZD-12	R-4	G-3	H-2
DJZD-13	R-5	G-3	H-3
DJZD-14	R-5	G-3	H-4
DJZD-15	R-5	G-4	H-5
DJZD-16	R-6	G-4	H-6
DJZD-17	R-6	G-4	H-7
DJZD-18	R-6	G-4	H-8
DJZD-19	R-6	G-4	H-9
DJZD-20	R-6	G-5	H-10
DJZD-21	R-7	G-5	H-1
DJZD-22	R-7	G-5	H-2
DJZD-23	R-7	G-6	H-3
DJZD-24	R-7	G-6	H-4
DJZD-25	R-7	G-6	H-5
DJZD-26	R-8	G-7	H-6
DJZD-27	R-8	G-7	H-7
DJZD-28	R-8	G-8	H-8
DJZD-29	R-8	G-8	H-9
DJZD-30	R-8	G-9	H-10

Note: Batches H1-H5 of ZBM were procured from Shaanxi Province; batches H6-H8 from Shandong Province; and batches H9-H10 from Sichuan Province. Batches G1-G4 of GOR were procured from Guangxi Province; batches G5-G9 from Yunnan Province. Batches R1-R8 of GRR were procured from Jilin Province.

### 2.2 Preparation of sample solutions and reference solutions

#### 2.2.1 Preparation of DJZD

Based on the prescription records of the Han Dynasty DJZD (Zhang, 2018), the formula was prepared using 10 g of ZBM, 55 g of ZOR, and 27.5 g of GRR. The botanical drugs were decocted with 800 mL of water by bringing the mixture to a boil over high heat (2000 W), followed by simmering at low heat (400 W) until the volume was reduced to 400 mL. The decoction was filtered through 200-mesh gauze, combined with 200 mL of maltose syrup, and concentrated to 300 mL at 600 W. The resulting solution was vacuum freeze-dried, finely ground, and stored as the final product.

For sample preparation, approximately 2 g of the freeze-dried DJZD powder was accurately weighed and extracted with 50 mL of methanol. The mixture was sonicated (250 W, 50 kHz) for 60 min, cooled to room temperature, and replenished with methanol to compensate for solvent loss. After shaking and filtration, 25 mL of the filtrate was collected, evaporated to dryness in a water bath, and the residue was reconstituted in methanol. The solution was transferred into a 10 mL volumetric flask, diluted to volume, mixed thoroughly, and filtered again.

All samples were filtered through a 0.22 μm nylon membrane (Millipore, United States) prior to analysis and stored at 4 °C until use.

#### 2.2.2 Preparation of standard solutions

Based on the preparation method established in a prior study from our laboratory (Xu et al., 2024), reference standard stock solutions were prepared as follows: hydroxy-α-sanshool, hydroxy-β-sanshool, and 6-gingerol were accurately weighed and individually dissolved in methanol to obtain solutions at a concentration of 2 mg/mL. Separately, ginsenosides Rb_1_, Rb_2_, Rb_3_, Rc, Rd, Re, Rf, and Rg_1_ were accurately weighed and dissolved in methanol to prepare solutions at 0.2 mg/mL.

Appropriate volumes from each of the eleven stock solutions were then combined and diluted with methanol to prepare a mixed reference standard solution with the following final concentrations: 0.2 mg/mL for hydroxy-α-sanshool, hydroxy-β-sanshool, and 6-gingerol; and 0.02 mg/mL for each of the ginsenosides (Rb_1_, Rb_2_, Rb_3_, Rc, Rd, Re, Rf, and Rg_1_).

All standard solutions were stored at 4 °C and filtered through a 0.22 μm nylon membrane (Millipore, United States) prior to analysis.

### 2.3 HPLC fingerprint analysis

#### 2.3.1 Chromatographic conditions

Analysis was performed using an LC-2030A high-performance liquid chromatograph (Shimadzu Corporation, Japan) equipped with a COSMOSIL 5C18-MS-II column (4.6 × 250 mm, 5 μm). The mobile phase consisted of acetonitrile (A) and water (B), eluted in a gradient as shown in Table 2 below; flow rate: 1.0 mL/min, detection wavelength: 203 nm, column temperature: 30 °C. The detection time was 120 min.

**TABLE 2:** Gradient elution conditions for fingerprint analysis.

Time (min)	A (%)	B (%)
0	19	81
35	19	81
55	29	71
70	29	71
100	40	60
115	19	81
120	19	81

#### 2.3.2 Method validation

The precision was determined by analyzing the same test sample (batch number: 20250507) in six consecutive injections. The stability was assessed by repeatedly analyzing the same test sample (batch number: 20250507) at 0, 4, 8, 12, 16, 20, and 24 h. Six independent test samples were prepared in parallel from the same batch of DJZD (Batch No.: 20250507) and analyzed in parallel to evaluate repeatability. For precision, stability, and repeatability analyses, ginsenoside Rd (peak 9) was used as the reference peak. The relative retention time (RRT) and relative peak area (RPA) of common peaks relative to the reference peak were calculated, along with the relative standard deviation (RSD) for both relative peak area and relative retention time (Table 3).

**TABLE 3:** Methodology validation results of fingerprint of DJZD.

Peak number	Accuracy RSD (%)		Repeatability RSD (%)		Stability RSD (%)	
	RRT	RPA	RRT	RPA	RRT	RPA
1	0.02	2.45	0.15	1.27	0.06	2.24
2	0.07	2.17	0.05	1.59	0.04	2.43
3	0.06	1.32	0.07	2.30	0.35	1.30
4	0.05	1.86	0.06	1.93	0.01	0.35
5	0.00	0.31	0.49	2.62	0.00	2.36
6	0.01	2.45	0.36	1.32	0.02	1.74
7	0.00	1.17	0.06	1.62	0.30	2.47
8	0.02	2.38	0.46	2.44	0.11	1.89
9	0.07	1.97	0.52	0.00	0.15	0.99
10	0.05	2.02	0.06	2.48	0.16	2.13
11	0.04	0.95	0.06	1.43	0.12	1.52

#### 2.3.3 Establishment of HPLC fingerprint profiles and similarity evaluation

Ten batches of ZBMs were randomly purchased from Shaanxi, Shandong, and Sichuan provinces; Nine batches of GORs were randomly purchased from Guangxi and Yunnan, and eight batches of GRRs were purchased from Jilin. These slices were randomly combined to obtain a total of 30 batches of DJZD. Following the method described in Section 2.1, DJZD test solutions were prepared. Analysis was conducted using the chromatographic conditions specified in Section 2.3. The resulting chromatograms were imported into the “Chinese Herbal Medicine Chromatographic Fingerprint Similarity Evaluation System (2012 Edition)” software for data analysis, generating HPLC overlay diagrams for all 30 batches. Using the chromatogram of DJZD-18 as the reference spectrum, spectral matching analysis was performed using the mean value method to calculate similarity values between the reference fingerprint and chromatograms of the 30 DJZD batches.

### 2.4 *In vitro* cellular experiments

RIN-14B cells were purchased from Shanghai Yaji Biotechnology Co., Ltd. and cultured in complete RPMI Medium 1,640 at 37 °C and 5% CO_2_. Cells in logarithmic growth phase were harvested for experiments when in optimal condition.

#### 2.4.1 Cytotoxicity of DJZD

Log-phase RIN-14B cells were seeded into 96-well plates at a density of 30,000 cells/mL (100 μL per well) and cultured in a 37 °C, 5% CO_2_ incubator. After cell attachment, supernatant was removed. Groups were established as follows: Con (control), Mod (medium), and DJZD treatment groups, each with 6 replicate wells. The Con group received cell-free, serum-free 1,640 medium; the Mod group received cell-containing, serum-free 1,640 medium; and the DJZD extract group received 1,640 medium supplemented with extracts at concentrations of 10, 25, 50, 100, 200, and 400 μg/mL. After 48 h incubation, supernatant was discarded. Ten microliters of CCK-8 solution was added, and the plate was shaken on a light-protected shaker for 2 h. Absorbance (A) at 450 nm was measured using a microplate reader. Cell survival rate (R) was calculated using Formula 1, and the IC50 value was determined.

$$R=\left(\text{AD }‐\text{ AB}\right)\;/\;\left(\text{AC }‐\text{ AB}\right)\times 100\%$$ (1)

AD is the absorbance of the DJZD group; AB is the absorbance of the blank group; AC is the absorbance of the control group.

Using the survival rate of RIN-14B cells exposed to different concentrations of DJZD as an indicator, screen for DJZD sample concentrations without cytotoxicity for subsequent 5-HT level experiments in cells. Compared to the Con group, the DJZD treatment group exhibited RIN-14B cell viability exceeding 90% at concentrations below 100 μg/mL. Therefore, the DJZD concentration was set to 100 μg/mL for subsequent experiments.

#### 2.4.2 ELISA assay to determine the effect of DJZD on 5-HT secretion levels in RIN-14B cells

RIN-14B cells in logarithmic growth phase were seeded into a 96-well plate at a density of 30,000 cells/mL, with 100 μL per well, and cultured in a 37 °C, 5% CO_2_ incubator. An appropriate amount of DJZD lyophilized powder was dissolved in 1,640 complete medium to prepare a 100 μg/mL drug-containing medium. Thirty batches of drug-containing medium (D1-D30) were prepared using the same method. After cell attachment, supernatant was removed and replaced with D1-D30 drug-containing medium, with three replicate wells per DJZD batch. Cultured at 37 °C in a 5% CO_2_ incubator for 24 h, cell supernatant was collected and 5-HT levels were measured using an ELISA kit according to the manufacturer’s instructions.

### 2.5 Spectro-efficacy analysis

Pearson correlation analysis, grey relational analysis, and partial least squares regression (PLSR)analysis were employed to investigate the spectro-efficacy relationship between the fingerprint spectrum of DJZD and 5-HT secretion levels in RIN-14B cells. Gray correlation analysis and PLSR analysis were performed using the online data analysis platform SPSS PRO, with the relative peak areas of common peaks across different batches of DJZD extract as the independent variable (X) and 5-HT levels as the dependent variable (Y). Pearson correlation analysis was conducted using SPSS.

### 2.6 The establish of rat model of POI

#### 2.6.1 The POI rats

All animal experiments were meticulously conducted in strict adherence to the institutional and national guidelines for the care and use of laboratory animals. Thirty SPF-grade male SD rats, aged 7 weeks, were purchased from Shandong Pengyue Biotechnology Co., Ltd. This study utilized 30 male SD rats (age: 6 weeks; weight: 280 ± 20 g). Rats were housed at 20 °C ± 2 °C under a 297 and 12 h light/dark cycle with free access to food and water. Five rats were housed per cage.

#### 2.6.2 The surgical operation

All rats were fasted for 24 h prior to surgery (with access to water). A 10% sodium pentobarbital solution was prepared for intraperitoneal anesthesia at a dose of 0.3 ml/100 g body weight. The rats’ limbs were secured to the operating table. After shaving the abdominal area, the site was disinfected with iodine solution and draped. A 2 cm midline incision was made in the lower abdomen. The skin and abdominal wall muscles were sequentially dissected. Using hemostatic forceps, the entire small intestine was gently extracted into the abdominal cavity and placed between two layers of moist gauze. A saline-moistened cotton swab was used to wipe the intestinal tract from the distal end upward toward the pyloric ring, repeating this process 6 times over approximately 10 min (taking care to avoid damaging mesenteric vessels). After friction, return the intestine to the abdominal cavity (ensuring it is straightened to prevent torsion). Close the abdomen with layered continuous suturing using 1–0 silk. After anesthesia wears off, withhold food but allow water intake; maintain body temperature. For the sham surgery group, do not manipulate the intestine after laparotomy; all other procedures are identical to the above.

#### 2.6.3 Grouping and dosing

In this study, 30 healthy male Sprague Dawley (SD) rats were randomly divided into five groups: Control Group (n = 6), DJZD pre-administration group (n = 6), model group (n = 6), DJZD post-administration group (n = 6), and sham-operated group (n = 6). Animals were housed in standard cages.

Experimental Medication: Based on historical and modern dosage measurements, literature review, and reference to contemporary clinical application doses, the original DJZD formula was modified to include 6 g ZBM, 12 g ZOR, 6 g GRR, and 30 g syrup.The commonly used clinical dosage of DJZD is 24 g/person/day (including 6 g ZBM, 12 g ZOR, 6 g GRR, crude herb medicine equivalent), and the dosage of 24 g/person/day for humans transferred to the rats was approximately 2.52 g/kg/day (crude herb medicine equivalent). In addition, the extraction yield of DJZD in our present study was approximately 15%, and therefore, the dose of water extracts of DJZD for rats was approximately 378 mg/kg. Consequently, we selected 378 mg/kg (0.378 g/kg) as our dose for the rat experiments.

Dosing Method: Rats in the DJZD Postoperative Administration group received oral administration of the decoction at 10 ml/kg body weight at 24 and 48 h post-surgery, totaling two doses. Rats in the DJZD Preoperative Administration group received oral administration at 10 ml/kg body weight once daily for 7 days prior to surgery, totaling seven doses. The DJZD control group, sham-operated group, and model group received drinking water at a volume of 10 ml/kg.

#### 2.6.4 General pharmacodynamic indicators

##### 2.6.4.1 Measurement of small intestinal propulsion rate in rats

Sixty minutes prior to euthanasia, all rats received 0.8 ml of 2% Evans blue dye via gastric lavage. After 60 min, laparotomy was performed, ligating the pylorus and terminal ileum. The entire small intestine was removed, laid flat on a saline-moistened dissection tray, and naturally straightened. Total small intestine length and the distance from the pylorus to the leading edge of blue dye were measured. Small Intestinal Propulsion Rate = (Distance from Pyloric Sphincter to Blue Edge/Total Small Intestinal Length) × 100%. The intact stomach was removed, and the masses of the whole stomach and gastric contents were measured separately. The gastric residue rate (%) was calculated using the formula: (Whole Stomach Mass - Washed Stomach Mass)/Whole Stomach Mass × 100%.

##### 2.6.4.2 Determination of gastric residue rate in rats

Completely remove the rat stomach. Measure the total stomach weight and gastric contents weight separately. Calculate the gastric residue rate (%) using the formula: (Total stomach weight - Weight of washed stomach)/Total stomach weight × 100%.

##### 2.6.4.3 Observation of small intestinal histopathological changes via HE staining

After euthanizing rats, approximately 3 cm of small intestinal tissue from the proximal cecum was harvested. Following dehydration, clearing, paraffin embedding, sectioning, and HE staining, the small intestinal tissue structure was examined under an electron microscope.

### 2.7 Metabolomics serum sample collection and pretreatment

A total of 54 serum samples underwent metabolomics analysis, including 6 blank samples, 6 sham-operated samples, 24 model samples (collected at 24 h, 36 h, and 48 h post-surgery), 6 DJZD post-administration samples (collected 24 h after the last dose), and 12 DJZD pre-administration samples (sampled preoperatively and 24 h postoperatively). Following collection, samples were centrifuged at 3,000 rpm for 10 min at 4 °C to separate serum, which was stored at −80 °C for serological indicator detection. Transfer 100 μL of serum sample into an EP tube, add 400 μL of 80% methanol aqueous solution; vortex, ice-bathe for 5 min, centrifuge at 15,000 g, 4 °C for 20 min; dilute a portion of the supernatant with mass spectrometry-grade water to achieve a methanol concentration of 53%; Centrifuge at 15,000 g, 4 °C for 20 min. Collect supernatant and inject into LC-MS for analysis (Want et al., 2013).

QC Samples: Pool equal volumes from each experimental sample as QC samples.

Blank Samples: Replace experimental samples with 53% methanol aqueous solution. Follow identical pretreatment procedures.

### 2.8 Non-targeted metabolomics analysis

#### 2.8.1 Chromatographic conditions

Hypersil Gold column (C18) (100 mm × 2.1 mm, 1.9 μm); mobile phase: 0.1% formic acid (A) – methanol (B), Gradient elution: 0–1.5 min, 98% A; 1.5–3 min, 98%–15% A; 3–10 min, 15%–0% A; 10–10.1 min, 0%–98% A; 10.1–12 min, 98% A. Column temperature: 40 °C; flow rate: 0.2 mL/min; Injection volume: 4 μL.

#### 2.8.2 Mass spectrometry conditions

Select scan range m/z 100–1,500; ESI source settings as follows: Spray voltage: 3.5 kV; Sheath gas flow rate: 35 psi; Auxiliary gas flow rate: 10 L/min; Capillary Temp: 320 °C; S-lens RF level: 60; Aux gas heater temp: 350 °C; Polarity: positive, negative; MS/MS secondary scanning: data-dependent scans.

#### 2.8.3 Bioinformatics analysis of metabolomics data

Raw data files were converted to mzXML format using ProteoWizard. Peak extraction and quantification were first performed with XCMS. Peak alignment across samples was achieved using retention time, mass-to-charge ratio, and other parameters. Peak area calibration was applied using the first sample to enhance quantification accuracy. Metabolite identification was performed by matching data against a high-quality secondary spectrum database, considering a 10 ppm mass tolerance and sum ions. Background ions were removed using blank samples. Raw quantitative results were normalized using the formula: raw sample value/(total metabolite value of sample/total metabolite value of first sample) to obtain relative peak areas. Data processing was conducted on a Linux operating system (CentOS version 6.6) using R and Python software. Specific packages and software versions are detailed in the results file readme.

Data analysis was performed via a cloud platform. Metabolites with VIP >1 and p < 0.05 were selected as potential differential metabolites, and t-tests were used to assess statistically significant differences between groups.

### 2.9 Statistical data analysis

SPSS 26.0 software was used for statistical analysis. Results are presented as mean ± standard deviation ( $\bar{x}$ ± s). Grey relational analysis and PLSR analysis were performed using the SPSSPRO website. GraphPad Prism 7.0 was employed for cell IC50 calculations and graphing. Identified metabolites were annotated using the KEGG database (https://www.genome.jp/kegg/pathway.html), HMDB database (https://hmdb.ca/metabolites), and LIPIDMaps database (http://www.lipidmaps.org/). HE staining results were visualized using CaseViewer 4.3 software. Graphs were generated using GraphPad Prism 10.0.

## 3 Results

### 3.1 Fingerprint methodology evaluation

#### 3.1.1 Precision

The relative standard deviation (RSD) of both the relative retention times and relative peak areas for all common peaks, using the ginsenoside Rd peak as the reference, were within 0.8% and 3.0%, respectively. These results confirm that the analytical instrument exhibited satisfactory precision.

#### 3.1.2 Repeatability

Method repeatability was assessed by analyzing six independently prepared samples. The RSD values for the relative retention times and relative peak areas of the common peaks (with ginsenoside Rd as reference) were ≤0.5% and ≤3.0%, respectively, indicating excellent repeatability of the sample preparation and analysis procedure.

#### 3.1.3 Stability

The stability of the test solution was evaluated over a 24 h period at room temperature. The RSD values for the relative retention times and relative peak areas of the common peaks (referenced to ginsenoside Rd) did not exceed 0.6% and 3.0%, respectively. This confirms that the sample solution remained stable throughout the analytical sequence.

### 3.2 Establishment of fingerprint profiles and similarity evaluation

**FIGURE 1:**
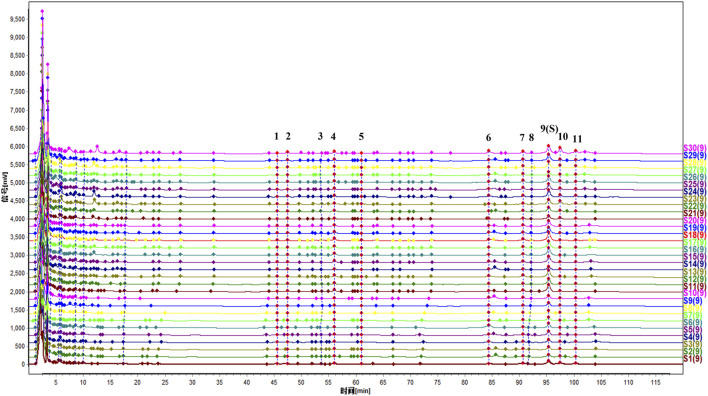
Overlay of fingerprint spectra for 30 batches of DJZD.

The characteristic HPLC fingerprint spectra of 30 batches of DJZD extract-s are shown in Figure 1. Fingerprint data were analyzed using the “Chinese Herbal Medicine Chromatographic Fingerprint Similarity Evaluation System (2012 Edition)” with a time window width of 0.1 min. Through multipoint calibration and Mark peak matching, 11 characteristic common peaks were calibrated.Si-milarity evaluation results between the reference fingerprint and chromatograms of each batch of DJZD extract showed that the similarity of all 30 randomly combined batches from different origins exceeded 0.9 (Table 4), indicating that the chemical characteristics of these 30 batches of DJZD were closely aligned.

**TABLE 4:** Similarity evaluation results of chromatograms for 30 batches of DJZD extracts.

Sample	Peak area										
No.	P1	P2	P3	P4	P5	P6	P7	P8	P9	P10	P11
DJZD-1	366069.8	490883.1	96717.4	1029051.4	25117.8	1000061.1	1169714.3	120001.9	6020695.5	1835237.9	1074458.4
DJZD-2	440670.8	338831.3	96861.2	959742.4	58634.2	1174843.6	1506943.0	144748.4	5939760.0	1990784.8	736903.1
DJZD-3	465469.2	353854.1	90812.1	968401.7	62403.9	1218377.6	1562096.9	152134.3	5956006.0	1212545.3	791316.9
DJZD-4	460001.4	526750.8	95105.0	1480331.0	69864.3	976402.3	1274699.6	134432.9	5130828.3	1249833.1	646872.4
DJZD-5	430558.7	583533.9	65157.6	1035271.7	60353.5	1043412.6	1545193.5	127066.0	5687418.5	1289571.0	740528.8
DJZD-6	591387.1	438588.8	90926.6	1085533.0	68612.6	1575756.0	1638137.9	138850.1	4839498.5	1276072.3	721223.9
DJZD-7	286871.2	403974.1	74812.5	1031794.0	69271.1	1785465.6	963960.9	127152.9	5086755.3	120826.8	766607.7
DJZD-8	423591.7	337243.7	87618.7	1474656.0	62592.0	1805678.2	1069749.9	151287.0	4141932.5	1459018.3	749411.7
DJZD-9	413373.5	359677.3	90675.8	1568928.1	65628.4	1879932.9	1879100.7	104187.2	5022517.5	1294860.1	785568.7
DJZD-10	415324.3	546009.4	90472.6	1472358.1	54159.7	1082370.5	1166920.9	104700.2	5573405.3	1573703.1	776776.9
DJZD-11	415140.3	738995.7	91178.6	1538433.1	84027.9	1403668.4	1800578.6	161554.1	8345197.0	1665916.6	524991.8
DJZD-12	486067.6	490005.4	141006.9	1268791.8	71426.7	1452541.2	981627.5	152883.1	6472659.0	1812097.5	504496.4
DJZD-13	418058.6	609664.9	94858.6	1141662.5	123507.0	1268855.8	1823195.3	173868.9	8702527.0	1523698.6	663219.6
DJZD-14	450849.6	414381.9	114521.1	1700219.8	68565.2	1534902.8	858729.9	127700.0	5824296.8	1517140.9	683191.3
DJZD-15	434110.2	506515.4	183408.7	1280346.1	76354.8	1231457.0	1180994.4	92299.2	7787882.0	1344101.6	772677.4
DJZD-16	488920.3	514912.8	126964.7	1781295.5	77621.3	1388909.6	1472593.9	142539.0	8936983.0	1936300.6	659174.6
DJZD-17	477584.7	544478.6	121503.4	1627298.4	76080.1	1265569.3	1419963.5	216738.6	8315169.0	1351223.4	677612.6
DJZD-18	468671.4	626912.1	117725.8	1685086.0	102265.4	1859444.4	1540758.0	161580.5	10303817.0	1460447.2	689546.2
DJZD-19	429288.9	467406.0	140347.9	1345378.1	64705.0	1773134.5	1150415.0	200355.9	7232096.5	1489530.2	681442.7
DJZD-20	437862.1	468870.7	143664.3	1125480.5	65428.7	1836498.5	829958.8	152939.4	6620685.5	1229297.7	712565.1
DJZD-21	498636.5	281471.6	90520.2	1512055.6	67652.4	1192151.5	1734185.7	112723.1	5079212.0	1885133.7	788240.1
DJZD-22	455288.1	316137.4	124698.4	935158.9	66245.7	1438342.1	1166221.8	162551.9	5003713.0	1964474.1	608219.5
DJZD-23	441068.7	397385.3	105147.9	949090.8	50473.0	852593.9	1311146.9	155101.2	5968330.0	1625763.8	767093.3
DJZD-24	40992.9	594574.6	150450.5	1121788.3	88439.0	1879417.6	1585549.8	174722.5	7867883.0	1510656.8	760992.3
DJZD-25	531839.1	535025.2	107996.6	977356.1	80806.5	1816324.3	2037437.6	172258.0	6235416.5	1416363.5	628498.4
DJZD-26	427361.8	679383.8	109961.6	1251763.9	66170.4	1590633.7	1950191.3	166825.2	7465873.5	1204720.5	709959.1
DJZD-27	359012.5	443200.7	107260.3	906157.0	63882.4	1848491.7	1265234.4	160585.4	6101367.0	1418784.6	688454.3
DJZD-28	463402.2	644937.4	102960.4	1035287.2	97569.5	1698223.6	1619377.5	179220.5	6722509.0	1727965.9	770534.5
DJZD-29	409355.5	665515.7	96210.8	1318555.3	62938.4	1314504.8	1764367.4	148755.8	7876668.5	1210651.9	603906.4
DJZD-30	443432.0	404967.9	92243.8	1244323.1	81243.5	1890573.5	1918787.5	130861.1	10058004.0	1264739.0	796143.8
RSD (%)	21.14	24.11	23.06	21.02	24.40	22.00	23.70	18.75	23.69	24.13	14.18

### 3.3 Identification of common peaks in fingerprint profiles

**FIGURE 2:**
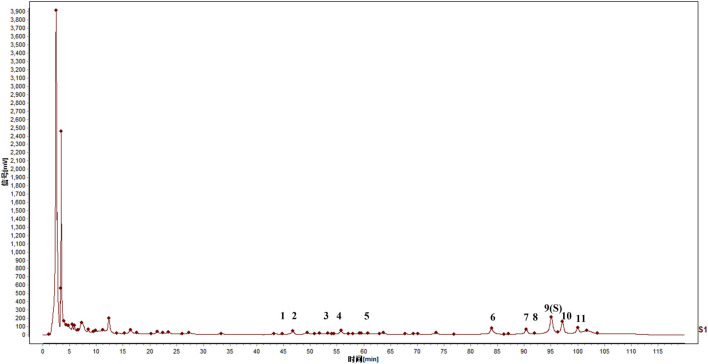
Identification of common peaks: 1, ginsenoside Rg_1_; 2, ginsenoside Re; 3, γ-Sanshool; 4, 10-gingerol; 5, ginsenoside Rb_1_; 6, ginsenoside Rc; 7, ginsenoside Rb_3_; 8, ginsenoside Rb_2_; 9, ginsenoside Rd (S); 10, hydroxy-α-sanshool; 11, hydroxy-β-sanshool.

Chromatographic analysis of freeze-dried powders from mixed reference standards and DJZD extract was conducted to identify common peaks in the fingerprint profiles. As shown in Figure 2, eleven common peaks were identified by comparing retention times with the reference standard: ginsenoside Rg_1_ (peak 1), ginsenoside Re (peak 2), ginsenoside Rb_1_ (peak 5), ginsenoside Rc (peak 6), ginsenoside Rb_3_ (peak 7), ginsenoside Rb_2_ (peak 8), ginsenoside Rd (peak 9), α-hydroxycinnamaldehyde (peak 10), and β-hydroxycinnamaldehyde (peak 11). First, the metabolites of the two common peaks were identified by comparing retention times and relative molecular masses with reference standards from the laboratory’s previously published articles (Xu et al., 2024). Subsequently, the remaining two metabolites were compared with the established information of 64 total metabolites and their relative retention times compared to reference standards, identifying the remaining two peaks as γ-Sanshool (Peak 3) and 10-gingerol (peak 4). Ultimately, 11 metabolites were identified.

### 3.4 Analysis of 5-HT level results

**FIGURE 3:**
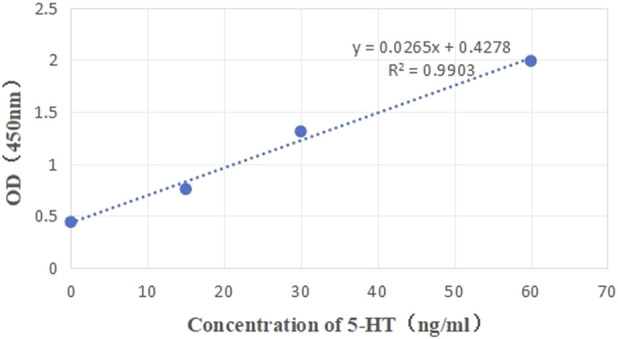
Standard curve for 5-HT.

A standard curve was plotted with standard concentration on the x-axis and corresponding OD values on the y-axis, as shown in Figure 3. The resulting equation was Y = 0.0265X + 0.4278. Using this equation, the concentration values for each sample were calculated, yielding the data presented in Table 5.

**TABLE 5:** Effect of 30 Batches of DJZD on Cellular 5-HT Secretion Levels ( $\bar{x}$ ±s, n = 3).

Samples	5-HT (ng/mL)
DJZD-1	11.98 ± 2.06
DJZD-2	11.57 ± 0.16
DJZD-3	10.62 ± 1
DJZD-4	13.37 ± 0.94
DJZD-5	11.2 ± 0.94
DJZD-6	11.82 ± 0.36
DJZD-7	8.85 ± 1.11
DJZD-8	8.27 ± 1.18
DJZD-9	10.7 ± 0.67
DJZD-10	9.5 ± 1.29
DJZD-11	9.32 ± 0.81
DJZD-12	10.47 ± 1.11
DJZD-13	9.33 ± 0.39
DJZD-14	9.76 ± 0.58
DJZD-15	9.19 ± 1.01
DJZD-16	8.31 ± 0.34
DJZD-17	10.36 ± 1.23
DJZD-18	8.61 ± 1.1
DJZD-19	8.84 ± 0.38
DJZD-20	9.33 ± 1.27
DJZD-21	8.15 ± 0.06
DJZD-22	9.52 ± 0.53
DJZD-23	7.89 ± 0.59
DJZD-24	8.88 ± 0.8
DJZD-25	9.93 ± 0.69
DJZD-25	8.51 ± 0.78
DJZD-27	8.03 ± 1.1
DJZD-28	10.1 ± 1.09
DJZD-29	9.55 ± 0.87
DJZD-30	7.97 ± 0.61

### 3.5 Study on spectrum-effect relationship

#### 3.5.1 Grey correlation analysis

Gray correlation analysis was performed using the SPSS PRO platform (https://www.spsspro.com/) (Scientific Platform Serving for Statistics Professional, 2021; Azzeh et al., 2010). Raw data underwent dimensionless processing, with 5-HT levels serving as the master sequence Y(k) and the peak areas of eleven common peaks in DJZD as the slave sequences Xi(k) (where i denotes peak number). The absolute difference sequence between master and sub-sequences was defined as Δi(k) = |Y(k) - Xi(k)|. The correlation coefficient was calculated as:

$$\text{Ai}\left(k\right)=\left[\Delta i\left(k\right)\min+\rho \times \Delta i\left(k\right)\max\right]/\;\left[\Delta i\left(k\right)+\rho \times \Delta i\left(k\right)\max\right]$$

The parameter ρ denotes the resolution coefficient, which was empirically set to 0.5 in this study (0 < ρ < 1). Based on the calculated association degree (γ), the correlation between a sub-sequence and the parent sequence was interpreted as follows: γ > 0.8 indicates strong association, 0.6 ≤ γ < 0.8 reflects moderate association, and γ < 0.6 suggests weak association (Shan et al., 2023).

The grey correlation results are presented in Table 6. As shown in Table 6, 11 peaks exhibit a correlation coefficient >0.8 with 5-HT level changes, with their correlation strengths ranked as follows: Peak 1 > 7 > 2 > 5 > 9 > 4 > 10 > 6 > 8 > 11 > 3. This indicates that the alteration of 5-HT levels in RIN-14B cells by the DJZD extract results from the combined effects of multiple metabolites.

**TABLE 6:** Correlation results between DJZD extract-induced 5-HT secretion levels in RIN-14B cells and peak areas of characteristic metabolites.

Relevance results		
Evaluation item	Relevance	Ranking
P1	0.908	1
P7	0.896	2
P2	0.884	3
P5	0.876	4
P9	0.873	5
P4	0.86	6
P10	0.843	7
P6	0.839	8
P8	0.832	9
P11	0.831	10
P3	0.815	11

#### 3.5.2 Partial least squares regression (PLSR) analysis

**FIGURE 4:**
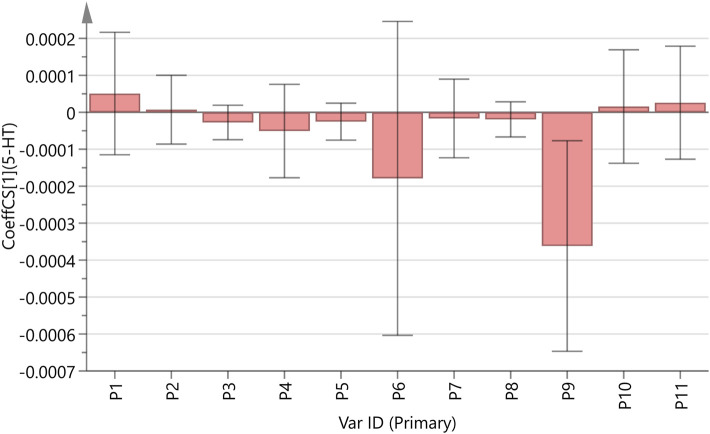
Regression coefficient diagram showing the relationship between the total peak area of DJZD and changes in 5-HT secretion levels in RIN-14B cells.

Using SIMCA14.1 software (version 14.1.02047), the peak areas of 11 common peaks across 30 batches of DJZD were standardized. These results served as independent variables (X), while the 5-HT secretion levels in cells after administration of each group were used as dependent variables (Y) for PLSR analysis, as shown in Figure 4. The results indicate that the established model parameters were R^2^X = 0.517 and Q^2^ = 0.0898. With R^2^X exceeding 0.5, the model demonstrated good fitting accuracy. In the fingerprint spectrum, except for peaks 1, 2, 10, and 11 which showed positive correlations with 5-HT levels, all other chromatographic peaks exhibited negative correlations with 5-HT levels.

#### 3.5.3 Variable importance in projection (VIP) analysis

**FIGURE 5:**
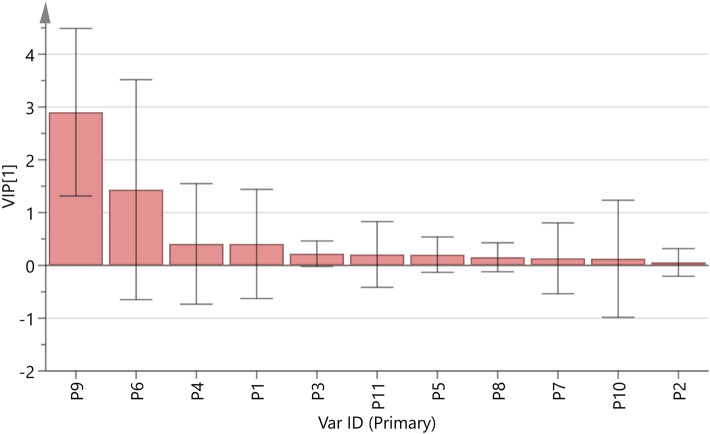
Projection importance analysis of DJZD total peaks and RIN-14B cell 5-HT secretion levels.

VIP analysis was performed based on the variable selection criterion within the PLSR framework. The VIP score quantifies the contribution of each independent variable in explaining the variance of the dependent variable (Li et al., 2022). Using SIMCA 14.1 software, a VIP plot (Figure 5) was generated by modeling the standardized peak areas of the common peaks from 30 batches of DJZD against corresponding cellular 5-HT secretion levels. The plot revealed that Peaks 9 and 6 exhibited the highest VIP scores, indicating their predominant influence on the variation in 5-HT secretion. This suggests that the metabolites corresponding to these peaks are likely the key bioactive constituents in DJZD responsible for modulating 5-HT levels, thereby contributing to its therapeutic effect against postoperative intestinal obstruction.

#### 3.5.4 Pearson Correlation Analysis

**FIGURE 6:**
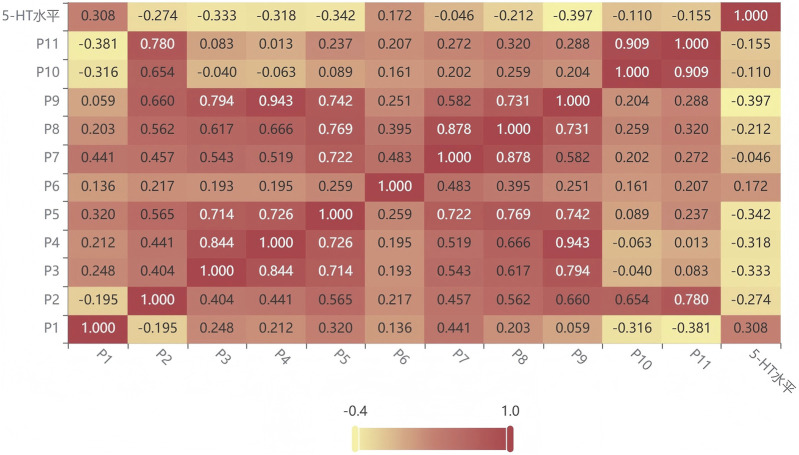
Pearson Correlation Analysis of Peak metabolites in DJZD and Changes in 5-HT Secretion Levels in RIN-14B Cells.

The results of Pearson correlation analysis are shown in Figure 6, where red and yellow represent positive and negative correlations, respectively. It can be seen that peaks 1 and 6 of DJZD are positively correlated with promoting 5-HT secretion in cells, while the others are negatively correlated with 5-HT secretion. This indicates that these active metabolites are the primary active metabolites in DJZD for treating postoperative intestinal obstruction.

### 3.6 General pharmacodynamic indicators

Following model establishment, surviving rats exhibited lethargy, arched backs, listlessness, and huddling behavior. Food intake and fecal output decreased markedly, with some rats producing virtually no feces.

#### 3.6.1 Intestinal transit rate

**FIGURE 7:**
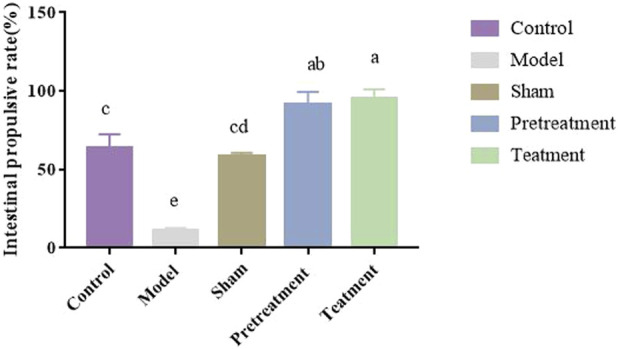
Intestinal propulsion rate in rats across groups. (Data are expressed as means ± SD. Different lowercase letters (a, b, c, d) indicate significant intergroup differences (P < 0.05), while identical letters denote no significant differences (P > 0.05); n = 6).

As shown in Figure 7, compared with the blank group, the small intestinal transit rate in rats after modeling was significantly reduced (P < 0.01). Compared with the model group, the sham-operated group exhibited a significantly higher small intestinal transit rate, indicating that intestinal transit impairment was indeed caused by the surgical manipulation of the intestinal tract. All DJZD administration groups showed increased small intestinal transit rates, with significant differences compared to the model group (P < 0.01).

#### 3.6.2 Gastric residue rate

**FIGURE 8:**
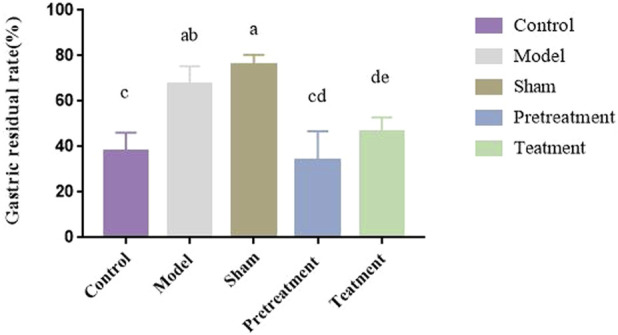
Gastric Residue Rate in Each Group of Rats (Data are expressed as means ± SD; different lowercase letters (a, b, c, d) indicate significant intergroup differences (P < 0.05), while identical letters (e.g., a and b) indicate no significant differences (P > 0.05); n = 6).

Gastric residual rates were calculated based on total and net gastric weights for each rat. Residual rates were visualized using GraphPad Prism software (Figure 8). Intergroup comparisons using SPSS software revealed statistically significant differences between the control group and the model group (P < 0.01). Comparisons between the model group and all drug-administered groups also showed statistically significant differences (P < 0.01), indicating that drug administration promoted gastrointestinal motility in rats, with the DJZD pre-administration group exhibiting the most pronounced effect.

### 3.7 Hematoxylin and eosin (HE) staining

**FIGURE 9:**
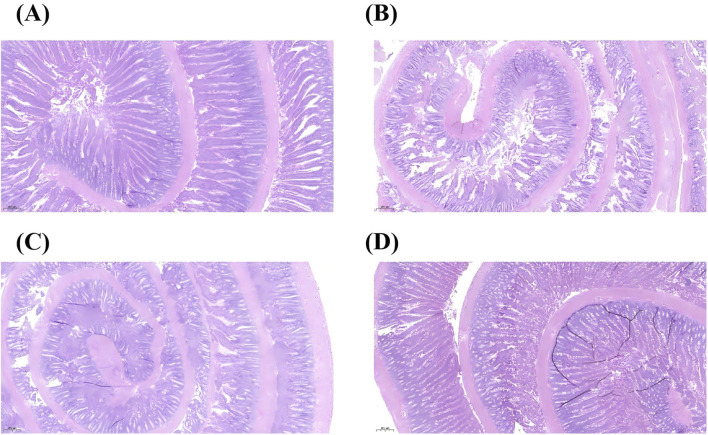
Histological changes in rat intestinal mucosa (HE staining, 5×).

As shown in Figure 9, in the control group, the intestinal mucosal epithelium exhibited orderly arrangement with well-defined villi and normal tissue structure, showing no signs of damage. In the model group, intestinal mucosal villi displayed disorganized arrangement, with most villi exhibiting uneven height, shortening, widened spacing, epithelial detachment, and interstitial congestion and edema. Some villi fused into patches, accompanied by inflammatory cell infiltration. Compared with the control group, the DJZD pre-administration group showed marked improvement in intestinal mucosal villi. Although arrangement was slightly irregular, villi thickness was largely uniform without fusion into patches. Interstitial congestion and edema improved relative to the control group, and inflammatory cell infiltration was significantly reduced. The DJZD post-administration group occasionally exhibited irregular villus arrangement and wider spacing, but showed significant improvement compared to the model group.

### 3.8 Metabolomics results

**FIGURE 10:**
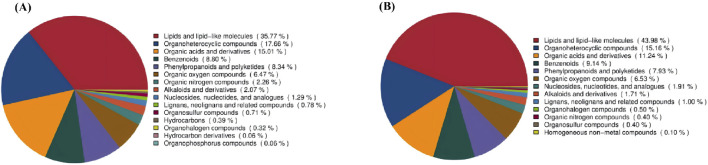
Metabolite Pie Charts **(A)** Metabolite pie chart in positive ion mode; **(B)** Metabolite pie chart in negative ion mode.

In this study, metabolites primarily comprised Lipids and lipid-like molecules (43.98%), Organoheterocyclic metabolites (15.16%), Organic acids and derivatives (11.24%), Benzenoids (9.14%), phenylpropanoids and polyketides (7.93%), and organic oxygen metabolites (6.53%) (Figure 10).

**FIGURE 11:**
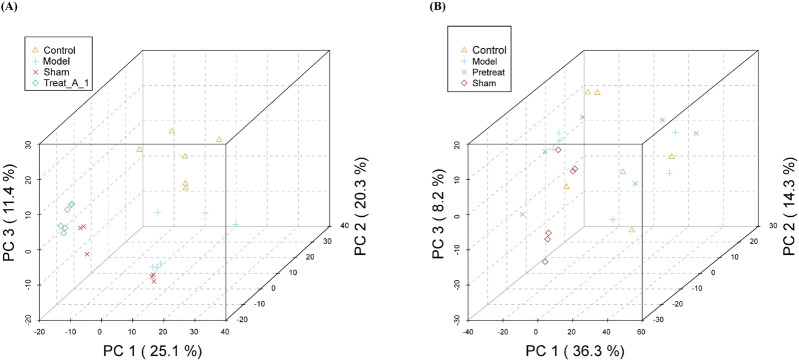
Three-dimensional principal compound analysis (PCA) score plot.

To characterize multivariate variations among samples across distinct experimental groups, principal compound analysis (PCA) was employed as a dimensionality-reduction tool to retain major variance information from the original high-dimensional dataset. As shown in Figure 11A, the distinct separation between the Control and Model groups in the PCA plot reflects significant metabolic changes induced by the model. The intermediate position of the Treat group indicates that the treatment partially modulated the metabolic perturbations. The overlap between sham and control groups suggests that the surgical procedure alone did not cause substantial metabolic shifts. These findings highlight the potential of the treatment to ameliorate model-induced metabolic dysregulation. However, the cumulative variance explained by the first three PCs was 56.8%, indicating that other factors may also contribute to the metabolic variations.

Figure 11B illustrated that model group’s samples diverged markedly along the positive PC1 axis, indicating profound metabolic disruption.The samples from DJZD post-administration group partially overlapped with model group but trended toward control group along PC1, suggesting metabolic restoration.The clear separation of model group from other groups validates the efficacy of disease induction. The intermediate position of DJZD pre-administration group implies a partial rescue of metabolic homeostasis, aligning with its proposed protective role. The high variance contribution of PC1 (36.3%) underscores its dominance in capturing disease-associated metabolic shifts. Future studies should identify key metabolites driving this separation.

**FIGURE 12:**
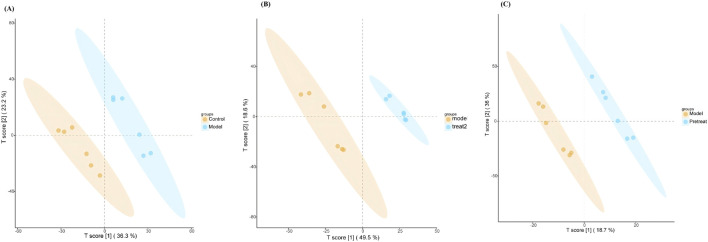
PLS-DA plot **(A)** Comparison between the control group and the model group **(B)** Comparison between the model group and the DJZD post-administration group **(C)** Comparison between the model group and the DJZD pre-administration group.

The overall difference in metabolites between each of the two groups was visualized by a Partial Least Squares Discriminant Analysis (PLS-DA) diagram. Figure 12 showed significant differences between the model group and the control group, with DJZD intervention causing the metabolites of POI rats to approach those of normal rats, a trend more pronounced in the DJZD pre-administration group.

**FIGURE 13:**
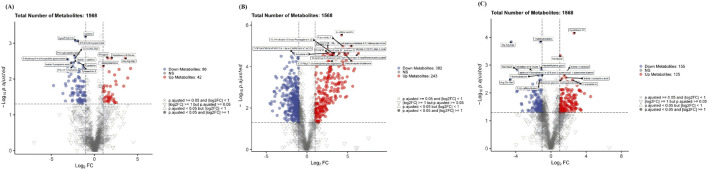
Volcano map **(A)** Comparison between the control group and the model group **(B)** Comparison between the model group and the DJZD post-administration group **(C)** Comparison between the model group and the DJZD pre-administration group.

The metabolite volcano plot (Figure 13) revealed that compared with the control group, the model group exhibited 42 significantly upregulated metabolites and 86 significantly downregulated metabolites. Compared with the model group, the DJZD pre-administration and DJZD post-administration groups showed 243 and 135 significantly upregulated metabolites, respectively, and 382 and 155 significantly downregulated metabolites, respectively. These results indicate that the DJZD post-administration group achieved greater reversal of pathological states in rats compared to the DJZD pre-administration. Detailed differences in metabolites between the model group and the DJZD pre-administration group are presented in Table 7. Detailed differences in metabolites between the model group and the DJZD post-administration group are presented in Table 8.

**TABLE 7:** Differential metabolites in serum between DJZD pre-administration group and model group.

Metabolites	t_R_/min	m/z	p.ajusted	FC	log2(FC)	Tendency
Gly Arg Asp	5.461	347.167	0.0001	0.0437	−4.5154	↓
1-O-Palmitoyl-2-O-acetyl-sn-glycero-3-phosphorylcholine	7.644	538.350	0.0136	22.7390	4.5071	↑
Sulfamethoxypyridazine	6.171	281.069	0.0090	0.0674	−3.8913	↓
Riboflavin	5.334	377.142	0.0015	0.0699	−3.8395	↓
Corticatic acid A	7.957	465.333	0.0105	13.9390	3.8011	↑
Fentanyl	8.167	337.225	0.0090	10.6320	3.4103	↑
Vitamin K1 oxide	8.476	467.349	0.0416	10.5830	3.4036	↑
7-Methyl-6, 8-bis(methylthio)pyrrolo (1, 2-a)pyrazine	6.085	225.049	0.0242	0.0976	−3.3577	↓
Arg Lys Leu	8.611	416.300	0.0261	0.1005	−3.3143	↓
(9Z, 11E, 13S)-13-hydroperoxyoctadeca-9, 11-dienoylcarnitine	6.889	456.331	0.0083	9.9120	3.3092	↑
2-Geranyl-3, 5-dihydroxybibenzyl	6.766	351.231	0.0377	9.6069	3.2641	↑
Mikanialactone	8.664	307.187	0.0442	0.1056	−3.2436	↓
7-Ketocholesterol	11.163	401.341	0.0083	9.2723	3.2129	↑
MG (0:0/22:4 (7Z, 10Z, 13Z, 16Z)/0:0)	6.754	429.295	0.0256	7.9437	2.9898	↑
Beta-Tocotrienol	7.624	411.325	0.0054	7.8410	2.9710	↑
14-Acetyltalatizamine	6.321	464.300	0.0355	7.3438	2.8765	↑
5-Hydroxy-6-methoxyindole glucuronide	5.406	340.102	0.0119	7.2758	2.8631	↑
Lovastatin	6.767	427.245	0.0256	7.2324	2.8545	↑
Astringin	6.144	429.117	0.0136	0.1407	−2.8294	↓
Quinaldic acid	5.452	196.037	0.0250	7.0139	2.8102	↑
Diacarnoxide C	10.326	409.330	0.0356	6.9852	2.8043	↑
Tormentic acid	7.072	511.339	0.0261	6.9758	2.8024	↑
Staplabin	6.322	486.282	0.0379	6.8570	2.7776	↑
Medicagenic acid	7.049	485.325	0.0342	6.4536	2.6901	↑
3-Methyldioxyindole	5.621	186.052	0.0119	6.3487	2.6665	↑
(+)-Valencene	6.769	227.179	0.0457	6.3147	2.6587	↑
Coronalolide methyl ester	7.232	497.323	0.0302	6.1348	2.6170	↑
Arg Phe Glu	7.294	451.230	0.0419	0.1646	−2.6029	↓
(22S, 23S)-28-homobrassinolide	10.512	517.350	0.0120	5.9839	2.5811	↑
Laprafylline	7.624	501.294	0.0136	5.9660	2.5768	↑
Gevotroline	7.934	310.168	0.0077	0.1711	−2.5472	↓
7-hydroxy-L-tryptophan	5.014	221.092	0.0256	5.7698	2.5285	↑
Lys Leu Ser	6.091	347.230	0.0440	0.1747	−2.5172	↓
LysoPE (0:0/18:4 (6Z, 9Z, 12Z, 15Z))	8.72	474.259	0.0165	5.7086	2.5131	↑
2-Methyl-3H-quinazolin-4-one	5.751	161.071	0.0231	5.4432	2.4445	↑
Sauchinone	7.908	379.113	0.0217	0.1850	−2.4346	↓
4-O-alpha-Cadinylangolensin	8.48	477.297	0.0302	5.3657	2.4238	↑
4, 4′-Diapolycopenedial	6.317	429.282	0.0077	5.3287	2.4138	↑
PGPC	9.335	610.370	0.0183	5.2949	2.4046	↑
Oxycodone	5.397	316.154	0.0380	5.1779	2.3724	↑
LysoPE (16:1 (9Z)/0:0)	8.719	452.276	0.0233	5.1711	2.3705	↑
17-Methyl-18-norandrosta-4, 13 (17)-dien-3-one	6.795	271.205	0.0220	5.0044	2.3232	↑
Multifidol glucoside	5.525	373.147	0.0256	5.0006	2.3221	↑
N-Docosahexaenoyl threonine	6.288	430.295	0.0355	4.9749	2.3147	↑
Anserine	1.353	241.129	0.0457	4.9462	2.3063	↑
Tyr Trp Gly	5.552	425.178	0.0380	4.8723	2.2846	↑
Butenoyl PAF	10.603	550.386	0.0090	4.8692	2.2837	↑
Auda	8.288	393.309	0.0122	0.2089	−2.2591	↓
Arg Phe Ala	7.928	393.224	0.0420	0.2106	−2.2477	↓
Bilobalide	5.508	309.094	0.0344	4.6766	2.2254	↑

**TABLE 8:** Differential metabolites in serum between DJZD post-administration group and model group.

Metabolites	t_R_/min	m/z	p.ajusted	FC	log2(FC)	Tendency
Tormentic acid	7.072	511.3391	0.0001	57.3630	5.8420	↑
2-Geranyl-3, 5-dihydroxybibenzyl	6.766	351.2313	0.0002	52.6990	5.7197	↑
PC(18:1 (11Z)/18:1 (12Z)-2OH(9, 10))	7.337	818.5840	0.0009	49.0730	5.6168	↑
Persicaxanthin	6.782	385.2728	0.0001	33.9830	5.0867	↑
Medicagenic acid	7.049	485.3255	0.0003	30.5520	4.9332	↑
LysoPA (0:0/18:2 (9Z, 12Z))	7.204	435.2476	0.0005	26.5520	4.7308	↑
5-Hydroxy-6-methoxyindole glucuronide	5.406	340.1023	0.0001	25.9060	4.6952	↑
18-Isovaleryloxygrindelic acid	6.738	421.2941	0.0010	23.8060	4.5732	↑
n-Pentadecylamine	6.798	228.2683	0.0060	0.0427	−4.5493	↓
Neoenactin NL2	7.971	387.2886	0.0001	23.2610	4.5398	↑
Anthosterone B	9.181	457.3280	0.0006	22.7290	4.5065	↑
Poricoic acid B	7.059	507.3077	0.0004	20.4570	4.3545	↑
Cordiachrome B	6.737	243.1375	0.0008	20.0260	4.3238	↑
24-Hydroxychiisanogenin	7.025	501.3200	0.0011	19.9370	4.3174	↑
Gamabufotalin	6.682	385.2354	0.0006	18.2830	4.1924	↑
Azadironol	7.015	523.3029	0.0027	17.1780	4.1025	↑
Heteroclitin D	6.839	505.1801	0.0016	0.0602	−4.0544	↓
Darutoside	8.498	507.2922	0.0048	0.0636	−3.9757	↓
Aikupikanyne A	6.764	251.1790	0.0006	15.6260	3.9659	↑
Arg Phe Ala	7.928	393.2240	0.0028	0.0650	−3.9443	↓
11beta-Hydroxyprogesterone	6.762	313.2156	0.0004	15.3120	3.9366	↑
4, 8 Dimethylnonanoyl carnitine	8.171	330.2632	0.0003	15.3060	3.9361	↑
2-Hexyl-3-phenyl-2-propenal	6.768	199.1479	0.0007	15.1960	3.9256	↑
L-Leucyl-L-Valine	5.301	231.1698	0.0016	14.4860	3.8566	↑
6-Oxopristimerol	7.356	481.2913	0.0003	0.0693	−3.8518	↓
Lucidone C	6.763	405.2626	0.0002	14.1160	3.8193	↑
4, 5-Dicaffeoyl quinic acid	6.839	517.1370	0.0020	0.0717	−3.8019	↓
2-Chlorooctadecanoic acid	6.78	319.2415	0.0013	13.4140	3.7457	↑
Dehydrofalcarinone	7.341	241.1564	0.0004	0.0762	−3.7144	↓
Vitamin K1 oxide	8.476	467.3492	0.0003	12.9950	3.6999	↑
Brassidic acid	11.586	321.3147	0.0011	0.0773	−3.6937	↓
16-Dehydroprogesterone	6.744	295.2053	0.0007	12.8250	3.6809	↑
Leupeptin	9.352	449.2866	0.0228	0.0792	−3.6581	↓
1-Formylneogrifolin	7.245	357.2444	0.0008	11.7320	3.5524	↑
1-O-Palmitoyl-2-O-acetyl-sn-glycero-3-phosphorylcholine	7.644	538.3497	0.0039	11.0900	3.4712	↑
Paeonenoide A	6.753	473.2866	0.0028	11.0550	3.4667	↑
3-Methyldioxyindole	5.621	186.0524	0.0004	10.5670	3.4016	↑
S-Methyl-L-methionine	7.331	181.1009	0.0007	10.4500	3.3854	↑
Arg Val Phe	8.607	421.2551	0.0103	0.0967	−3.3698	↓
Trifolirhizin	6.148	469.1102	0.0014	0.0987	−3.3411	↓
3beta-Fluoro-5beta-pregnan-20-one	8.057	321.2570	0.0002	9.8204	3.2958	↑
Dukunolide B	6.84	499.1629	0.0057	0.1103	−3.1803	↓
Corticatic acid A	7.957	465.3334	0.0003	8.9474	3.1615	↑
Pentosidine	7.32	379.2082	0.0003	0.1172	−3.0926	↓
Hexadeca-4, 7, 10, 13-tetraenoic acid	8.074	249.1825	0.0011	0.1207	−3.0510	↓
Cordycepin	6.854	274.0902	0.0028	0.1207	−3.0504	↓
Talatisamine	11.595	422.2934	0.0010	0.1209	−3.0486	↓
Bis(ethylphenyl)ether	7.333	227.1426	0.0004	8.2219	3.0395	↑
delta9-Tetrahydrocannabinol hemisuccinate	7.246	415.2481	0.0045	8.2020	3.0360	↑
5-epi-smenospongiarine	6.652	414.2995	0.0010	8.1679	3.0300	↑

**FIGURE 14:**
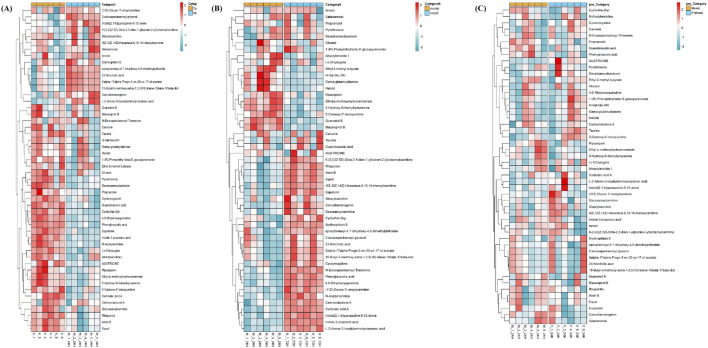
Heat map of metabolite hierarchical clustering **(A)** Comparison between the control group and the model group **(B)** Comparison between the model group and the DJZD post-administration group **(C)** Comparison between the model group and the DJZD pre-administration group.

To provide an intuitive visualization of intersample relationships and expression differences of metabolites across groups, as well as to directly observe metabolic variations between groups, a hierarchical clustering heatmap was constructed based on the identified differential metabolites (Figure 14).

The heatmap revealed distinct metabolic profiles between the Model and DJZD pre-administration groups. Metabolites such as Austicine, Pyrethrosine, and Olivetol showed significantly higher expression in the Model group, whereas Taurine and Cedronolactone A were more abundant in the DJZD pre-administration group. Clustering analysis indicated that Model samples formed a tight cluster, suggesting consistent metabolic responses, while DJZD pre-administration group’s samples exhibited greater heterogeneity. Notably, several bioactive metabolites including Indole-3-propionic acid and Stearoylcarnitine displayed time-dependent accumulation patterns, indicating potential involvement in stress response or energy metabolism.

Notably, several metabolites such as Austicine, Rhapontin, Eupatorin, and L-2-Amino-3-(oxalylamino)propionic acid exhibited significantly higher abundance in the treatment group. In contrast, metabolites like Olivetol and Decarbamoylsaxitoxin were more abundant in the model group. The clustering pattern indicated that DJZD treatment induced substantial metabolic reprogramming.

### 3.9 Metabolic pathway analysis

To further explore the interrelationships among metabolites, the identified differential metabolites were imported into the KEGG database for metabolic pathway enrichment, resulting in metabolic pathway bubble charts.

**FIGURE 15:**
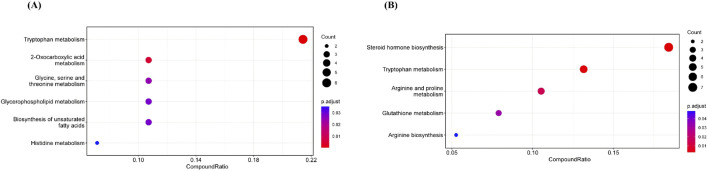
Metabolic pathway analysis of potential biomarkers.

As shown in Figure 15A, compared with the control group, the model group identified metabolic pathways significantly associated with postoperative intestinal obstruction, primarily including tryptophan metabolism, 2-oxocarboxylic acid metabolism, glycine, serine, and threonine metabolism, glycerophospholipid metabolism, biosynthesis of unsaturated fatty acids, and histidine metabolism. These pathways may be involved in the pathogenesis of postoperative obstructive injury. The CompoundRatio for the tryptophan metabolism pathway was the highest (approximately 0.22) among the differentially enriched metabolites. It also exhibited the largest bubble (Count = 6) and the reddest color (p.adjust ≈0.01), indicating that this pathway shows the most significant enrichment of differential metabolites and may play a central role in metabolic changes within the study system.

As shown in Figure 15B, compared to the model group, enriched pathways in the treatment group primarily included: Steroid hormone biosynthesis, Tryptophan metabolism, Arginine and proline metabolism, Glutathione metabolism Tryptophan metabolism contained the highest number of differential metabolites (Count = 7, p.adjust <0.05), while steroid hormone biosynthesis was the least significant (p.adjust ≈0.04). The high metabolite coverage in tryptophan metabolism suggests its role as a regulatory hub.

## 4 Discussion and conclusion

The therapeutic effects of traditional Chinese medicine formulas are attributed to the synergistic actions of multiple bioactive metabolites, and deciphering the material basis and efficacy correlation is the core of modern pharmacological research on classical formulas. In this study, three spectro-efficacy analysis methods were integrated to systematically explore the anti-POI effect of DJZD, and the results confirmed that its therapeutic effect is a consequence of the collaborative action of multiple metabolites in the formula. Among them, ginsenoside Rc (Peak 6) and ginsenoside Rd (Peak 9) were identified as the primary bioactive constituents, which not only clarifies the key material basis for DJZD against POI but also provides a targeted reference for the quality control and efficacy evaluation of the formula. Given the inherent differences in chemical composition and therapeutic efficacy of DJZD samples from different origins and batches caused by factors such as medicinal material quality and preparation processes, we adopted a pharmacophore based research strategy, combined the separation advantage of HPLC technology with the statistical advantage of correlation analysis, and successfully distinguished the chemically distinct metabolites among different samples. Furthermore, PLSR analysis was applied to correlate the HPLC fingerprint of DJZD with the 5-HT level efficacy indicator, which quantitatively reflected the contribution of each chemical metabolite to the anti-POI effect, and established a direct “metabolite-efficacy” correlation for DJZD, laying a foundation for the precise interpretation of its pharmacological mechanism. However, the Q^2^ value (0.0898) of the model in this study falls below the ideal threshold, indicating limited predictive capability of the current model (Luo et al., 2024; Zhu et al., 2022). This may be attributed to the small sample size and variability in certain efficacy indicators. Therefore, this result should only serve as preliminary exploratory reference for the component-activity relationship. Further validation requires expanding the sample size and employing more systematic analytical methods, such as orthogonal partial least squares discriminant analysis.

The concept of “preventing disease before its onset” is the core of traditional Chinese medicine (TCM) health preservation and disease treatment, which emphasizes timely intervention in the pre-disease stage—the progressive phase of pathological development where pathological changes are reversible with targeted regulation (Liu et al., 2014; Hao et al., 2024). However, the pre-disease stage is characterized by dynamic complexity and subtle pathological changes, and its biological basis and evolutionary patterns remain unclear to date, which severely restricts the quantitative characterization, precise identification and early clinical intervention of pre-disease states. To explore the intervention effect of DJZD based on the TCM “preventive treatment” theory, we innovatively established a DJZD pre-administration group (healthy rats were given intragastric DJZD for 7 days before POI modeling) and a conventional post-administration group, and compared the recovery process of POI in rats with the two intervention modes. Pharmacodynamic evaluation showed that rats in the model group exhibited typical POI symptoms such as lethargy, hypoactivity, hunchback, and a significant reduction in food intake and fecal output, which are consistent with the pathological characteristics of gastrointestinal motility disorder and digestive function decline after abdominal surgery. In contrast, both the pre-administration and post-administration groups showed varying degrees of improvement in the above abnormal symptoms: food intake and fecal output were significantly increased, and the symptoms of lethargy and hunchback were completely relieved. Gastric emptying rate and intestinal propulsive rate are important indicators reflecting the recovery of gastrointestinal motor function; the results showed that the small intestinal propulsive rate of all DJZD-treated groups was significantly higher than that of the model group (P < 0.01), and the pre-administration group exhibited the most prominent improvement effect. These pharmacodynamic results fully confirm that both pre- and post-administration of DJZD can effectively intervene in the progression of POI and promote the recovery of gastrointestinal function, and the pre-administration intervention based on the “preventing disease before its onset” theory shows a superior therapeutic effect, which provides experimental evidence for the clinical application of TCM preventive treatment in POI. On this basis, metabolomics analysis was further conducted to explore the potential differences in metabolic regulatory pathways of DJZD for POI treatment under different administration time windows, aiming to reveal the molecular mechanism of the superior efficacy of pre-administration from the perspective of metabolic reprogramming.

Metabolomics, as a core technology of systems biology, can comprehensively reflect the dynamic changes of endogenous metabolites in organisms under pathological and drug intervention conditions, and is an effective means to decipher the metabolic regulatory mechanism of TCM formulas (He et al., 2024; Jin and Zhao, 2025). In this study, metabolomics analysis identified the key metabolic pathways involved in the treatment of POI by DJZD pre- and post-administration, including steroid hormone biosynthesis, tryptophan metabolism, 2-oxoglutarate metabolism, glycine-serine-threonine metabolism, glycerophospholipid metabolism and unsaturated fatty acid biosynthesis, which clarifies the multi-pathway metabolic regulatory characteristics of DJZD against POI. Tryptophan, as an essential amino acid, is not only a raw material for protein synthesis but also a key precursor for the synthesis of a variety of bioactive substances, among which 5-HT is a crucial neurotransmitter and intestinal regulatory factor involved in the regulation of gastrointestinal motility (Bai et al., 2022), mucosal barrier function (Zhang et al., 2025) and mood regulation (Zeng et al., 2024). The significant enrichment of the tryptophan metabolic pathway in the differentially expressed metabolites suggests that the metabolic reprogramming of this pathway is a core link in the metabolic disorder of POI, and DJZD may regulate the synthesis and metabolism of 5-HT through intervening in tryptophan metabolism, thereby exerting the anti-POI effect. 2-oxoglutarate is a key intermediate of the tricarboxylic acid (TCA) cycle, which is the core pathway of cellular energy metabolism; its significant enrichment indicates that POI is accompanied by the remodeling of cellular energy metabolism, and DJZD may regulate the metabolic flux of TCA cycle intermediates to meet the energy demand of gastrointestinal tissue repair and functional recovery (Ye et al., 2021). Glycine, serine and threonine metabolism is the main source of one-carbon units in organisms, which provides essential raw materials for nucleic acid synthesis, protein methylation and other biological processes; the enrichment of this pathway suggests that DJZD may promote the proliferation and differentiation of gastrointestinal mucosal epithelial cells through regulating one-carbon metabolism, and participate in the epigenetic regulation of related functional genes, thus accelerating tissue repair. Glycerophospholipids are the basic metabolites of biological membranes, and their metabolic changes directly affect membrane fluidity, signal transduction and cell barrier function (Xu et al., 2026); the regulation of glycerophospholipid metabolism by DJZD may be an important mechanism to maintain the integrity of intestinal mucosal barrier and improve the abnormal signal transduction of gastrointestinal smooth muscle cells. Unsaturated fatty acid biosynthesis is closely related to the structural stability of cell membranes and the regulation of inflammatory responses; DJZD may intervene in the inflammatory cascade reaction of POI and maintain the normal physiological function of gastrointestinal cells by regulating the metabolism of unsaturated fatty acids. In contrast, histidine metabolism showed relatively low enrichment in the study, suggesting that it is not a core metabolic pathway in the POI pathological process and DJZD intervention system, but as an amino acid containing an imidazole group, histidine is the precursor of histamine (a key mediator involved in intestinal smooth muscle contraction and inflammatory response), thus it may still play a minor regulatory role in specific physiological and pathological links of POI.

In summary, this study reveals that the mechanism of DJZD in improving POI is multi-faceted and multi-targeted, and the core regulatory mechanisms are mainly reflected in two aspects. First, DJZD enhances the intestinal barrier function to alleviate POI: during the occurrence and development of POI, intestinal mucosal damage is an important pathological link that exacerbates intestinal inflammatory response and motility disorder. 5-HT can regulate the repair of intestinal mucosa by activating 5-HT_3_ receptors to promote the proliferation of mucosal epithelial cells; DJZD can upregulate the level of intestinal 5-HT, thereby indirectly activating 5-HT_3_ receptors, accelerating the repair of damaged intestinal mucosal epithelial cells, reducing intestinal bacterial translocation and the release of inflammatory factors, and ultimately forming a positive feedback loop of “motility repair - mucosal protection” to reverse the pathological state of POI. Second, DJZD regulates the balance of endogenous metabolites to intervene in POI: by targeting and regulating multiple metabolic pathways such as tryptophan and phenylalanine, DJZD can reduce the production of pro-inflammatory metabolites in the body, while increasing the content of beneficial metabolites such as L-tyrosine that promote intestinal tissue repair and inflammatory resolution, thereby relieving the inflammatory response and motility disorder of the gastrointestinal tract, and exerting a significant therapeutic effect on POI.

Notably, this study confirmed the superior anti-POI effect of DJZD pre-administration based on the TCM “preventing disease before its onset” theory, and its mechanism may be related to the early regulation of the body’s metabolic network and the pre-activation of the intestinal mucosal protection and motility regulation system, which avoids the further deterioration of pathological damage after modeling. This finding not only expands the clinical application of DJZD in POI but also provides a new research idea for the modernization of TCM preventive treatment theory. However, this study still has certain limitations: the specific regulatory mechanism of DJZD on the key enzymes and transporters in the identified metabolic pathways remains to be further verified; the molecular target of the core metabolites (ginsenoside Rc, ginsenoside Rd) in the anti-POI effect needs to be further explored. This study preliminarily reveals that DJZD may exert its effects by regulating the tryptophan pathway. In subsequent work, we will employ transcriptomics, protein immunoblotting, and cellular molecular biology techniques to validate core targets such as 5-HT_3_R, 5-HT_4_R, and TPH-1, thereby fully elucidating its molecular mechanisms.

## Data Availability

The original contributions presented in the study are included in the article/supplementary material, further inquiries can be directed to the corresponding authors.
